# Analysis of Under-Diagnosed Malignancy during Fine Needle Aspiration Cytology of Lymphadenopathies

**DOI:** 10.3390/ijms241512394

**Published:** 2023-08-03

**Authors:** Jeeyong Lee, Hwa Jeong Ha, Da Yeon Kim, Jae Soo Koh, Eun Ju Kim

**Affiliations:** 1 Division of Radiation Biomedical Research, Korea Institute of Radiological and Medical Sciences, Seoul 01812, Republic of Korea; jeeyongl@gmail.com (J.L.); kimd1106@daum.net (D.Y.K.); 2 Department of Pathology, Korea Cancer Center Hospital, Korea Institute of Radiological and Medical Sciences, Seoul 01812, Republic of Korea; cytoha@kirams.re.kr (H.J.H.); jskoh@kirams.re.kr (J.S.K.); 3 Convergence Institute of Biomedical Engineering and Biomaterials, Seoul National University of Science and Technology, Seoul 01811, Republic of Korea; 4 Department of Radiological and Medico-Oncological Sciences, University of Science and Technology, Daejeon 34113, Republic of Korea; 5 Institute for Molecular Bioscience, The University of Queensland, Carmody Rd., St Lucia, Brisbane, QLD 4072, Australia

**Keywords:** lymphadenopathy, fine needle aspiration cytology (FNAC), under-diagnosis, malignancy, biomarker

## Abstract

Fine needle aspiration cytology (FNAC) is a useful tool in the evaluation of lymphadenopathy. It is a safe and minimally invasive procedure that provides preoperative details for subsequent treatment. It can also diagnose the majority of malignant tumors. However, there are some instances where the diagnosis of tumors remains obscure. To address this, we re-analyzed the misinterpreted patients’ samples using mRNA sequencing technology and then identified the characteristics of non-Hodgkin’s lymphoma that tend to be under-diagnosed. To decipher the involved genes and pathways, we used bioinformatic and biological analysis approaches, identifying the response to oxygen species, inositol phosphate metabolic processes, and peroxisome and PPAR pathways as possibly being involved with this type of tumor. Notably, these analyses identified FOS, ENDOG, and PRKAR2B as hub genes. cBioPortal, a multidimensional cancer genomics database, also confirmed that these genes were associated with lymphoma patients. These results thus point to candidate genes that could be used as biomarkers to minimize the false-negative rate of FNAC diagnosis. We are currently pursuing the development of a gene chip to improve the diagnosis of lymphadenopathy patients with the ultimate goal of improving their prognosis.

## 1. Introduction

Lymphadenopathy, one of the most common symptoms among clinical presentations of patients [1], can be indicative of a number of diseases, including reactive lymphoid hyperplasia, infectious disease, granulomatous lymphadenitis, metastatic malignancy, and primary lymphoproliferative disorder. Lymphadenopathy occurs where lymph nodes are enlarged, for instance, cervical, axillary or supraclavicular areas. Multi-site lymphadenopathy is usually an indicator of a more serious disease. Perhaps the most concerning is the possibility that an unexplained lymphadenopathy could be related to a higher chance of an underlying malignancy [2,3].

For unexplained lymphadenopathy, the cause could be determined by a tissue biopsy—the most reliable diagnostic method—but this is difficult to apply in all patients. Because of difficulties associated with tissue biopsies, fine needle aspiration cytology (FNAC), a fast and cost-efficient method, has been used for many years as a first-line procedure for differentiating malignancy from other causes [4]. However, there are instances where under-diagnosis could occur with FNAC. For example, the tumor could be heterogeneous, with only a small part being malignant and the rest benign. Other cases of under-diagnosis are attributable to a pathology error in which the malignant features of the tumor are obscure. Notably, FNAC as a diagnostic procedure can produce high false-negative rates in patients with primary lymphoproliferative disorder [5]. Immunocytochemistry could lessen these difficulties and support a cyto-morphological interpretation [6,7]. However, there is still room for improvement in cases where the classification of the primary tumor remains obscure [8,9].

In this study, we sought to improve the diagnoses of obscure cases by focusing on cases where errors in pathology occurred during FNAC. To this end, we performed both FNAC and tissue biopsy in patients who had been diagnosed as benign based on FNAC. A comparison of the respective diagnoses identified five mismatched patients who were later diagnosed as malignant by tissue biopsy. We then processed the corresponding mismatched biopsy tumors for RNA sequencing together with matched samples as controls. After obtaining an expression profile for each group, we used bioinformatics and biological analysis methods to analyze the candidate genes and pathways that could lead to under-diagnosis with FNAC. We hope that the biomarker candidates identified here will increase the reliability of FNAC and ultimately improve the quality of life of patients after treatment.

## 2. Results

### 2.1. Comparison of FNAC and Tissue Biopsy

To investigate the reliability of FNAC, we assessed 432 multiple lymphadenopathy patients (247 males and 185 females) at KCCH. The mean age of patients was 58.2, and the average size of their lymphadenopathy was 1.9 cm. Patient FNAC diagnoses were classified as follows: metastatic carcinoma, 44.4%; benign, 35.9%; atypical cells, 9%; malignant lymphoma, 5.1%; and suspicion of malignancy, 2.1%. In 3.5% of cases, there was insufficient material for diagnosis [10].

**Figure 1:**
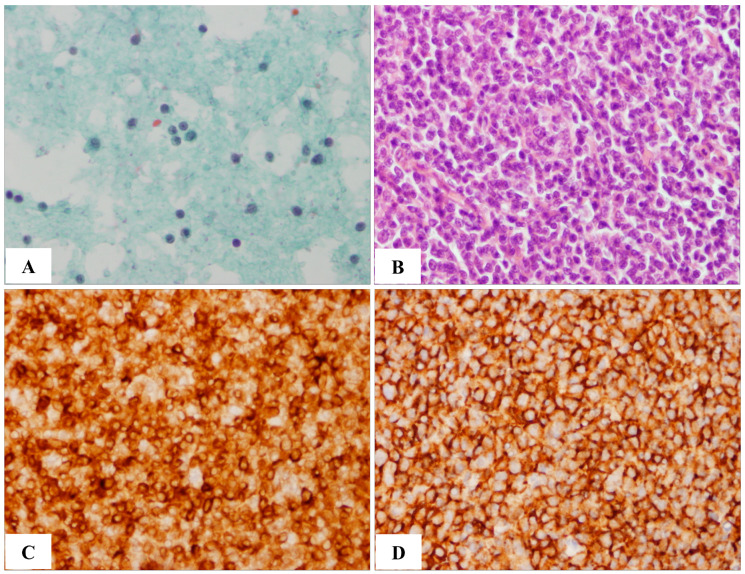
Comparison of cytology and histology. An under-diagnosed case initially diagnosed by FNAC as reactive hyperplasia, but corrected to follicular lymphoma (grade 1) with histological diagnosis, is shown. (**A**) Pap smear showing small lymphoid cells. (**B**) H&E-stained histologic section. (**C**,**D**) Immunohistochemistry (IHC) showing positive staining for BCL2 (**C**) and CD20 (**D**). 400× magnification.

Because false-positive cases (over-diagnosis) would typically proceed to a tissue biopsy step, where they could be corrected, we focused on false-negative cases (under-diagnosis). Tissue biopsies were subsequently performed on cases diagnosed by FNAC as benign. A comparison of respective pairs of diagnoses (*n* = 155) revealed two types of under-diagnosed cases. The first category (Cat-I) was initially diagnosed as benign and then was changed to metastatic cancer (*n* = 2); in the second category (Cat-II), the diagnosis was changed from benign to non-Hodgkin’s lymphoma (NHL) (*n* = 5). Upon careful re-evaluation, we concluded that under-diagnosis in Cat-I was attributable to the heterogeneous structure of the tumor, where a small part of the tumor was malignant and the rest was benign. On the other hand, Cat-II showed no obvious error during FNAC. Pap smears (Figure 1A) and H&E staining (Figure 1B) showed no signs of malignancy. However, immunohistochemistry showed positive staining for BCL2 (Figure 1C) and CD20 (Figure 1D), which was consistent with NHL. Therefore, the Cat-II under-diagnoses highlight the insufficiencies of the current FNAC procedure and underscore the need for these procedures to be upgraded to reduce false-negative diagnoses.

### 2.2. RNA-Seq Analysis of Under-Diagnosed Patients’ Samples

To minimize Cat-II false-negative results, we investigated the characteristics of under-diagnosed NHL. All patients showed similar lymphadenopathic symptoms (see Table 1 for patients’ characteristics). Aspirated samples from under-diagnosed patients were processed as described in “Materials and Methods”. mRNA was extracted from the patient samples (Cases 1–4), sequenced, and used to generated gene expression profiles. Samples from benign patients were also processed as controls (Cases 5 and 6).

A total of 27,685 genes were analyzed in each patient sample. First, we performed a gene set enrichment analysis (GSEA) [11] (Table 2), which provides information on changes in the involvement of various metabolic pathways. Interestingly, cytochrome p450 and PPAR signaling pathways were among the pathways involved, providing insight into the underlying molecular characteristics of NHL.

**Figure 2:**
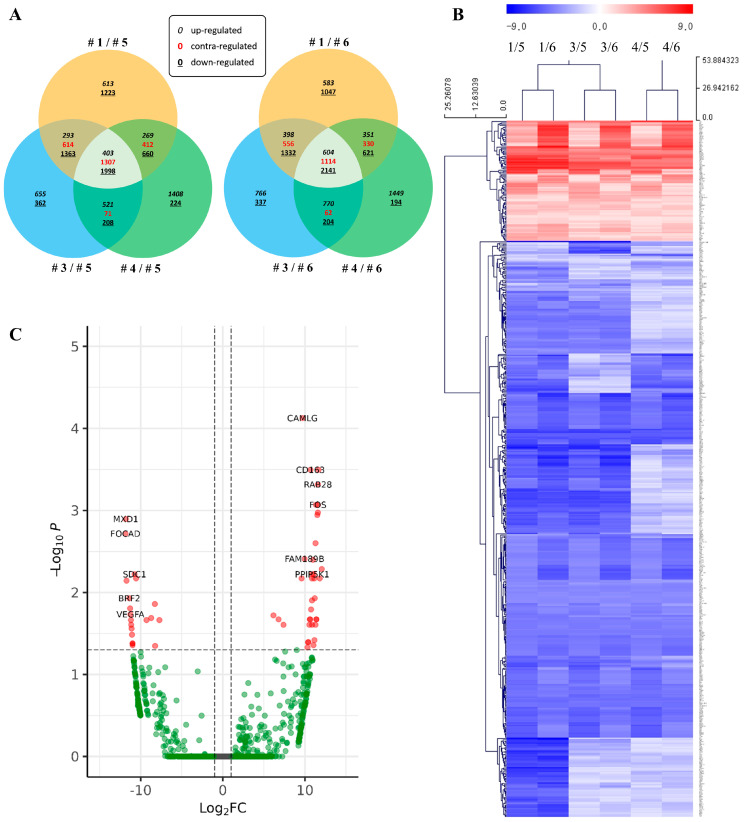
RNA sequencing and analysis. (**A**) Venn diagrams of DEGs in NHL patient samples (Cases 1–3) compared with benign patient samples (Cases 5–6) show the number of DEGs. (**B**) Heatmap of genes that are significantly differentially expressed between NHL and benign patient samples. Representations of genes were processed using the general linear model likelihood ratio test (*p* < 0.05 and absolute log_2_ fold change >1). (**C**) Volcano plot showing DEGs.

Genes were filtered for differentially expressed genes (DEGs) using the criteria, raw *p*-value < 0.05 and absolute value of fold change (|FC|) ≥ 2, and they were depicted as a scatter plot (Figure 2A). Venn diagram representations of filtered DEGs showed significant variations among patients’ samples; thus, the genes were further sorted based on intersections of the Venn diagram in common for all three samples. Three patient samples (Cases 1, 3 and 4) exhibited 3708 DEGs in common with Case 5 and 3859 DEGs in common with Case 6. After a comparison of these common DEG sets, 544 DEGs that were contained within both sets were selected for subsequent bioinformatic analysis.

### 2.3. Functional Classification of DEGs Associated with Under-Diagnoses

The Multi-experiment viewer (MeV) was used to generate a heatmap of the selected 544 DEGs. The heatmap analysis [12] showed the clustering of DEGs in the NHL patient sample, independent of controls (Figure 2B), confirming the consistency of our dataset between patients. To identify DEGs, we also generated a volcano plot (Figure 2C). We also performed a gene ontology (GO) analysis [13], annotated by the DAVID database (https://david.ncifcrf.gov/, accessed on 15 September 2022), to obtain an overview of the function of protein products of our DEGs (Table 3). The GO properties most frequently associated were integrin-mediated signaling pathways (biological process), mitochondria (cellular compartment), and protein tyrosine phosphatase activity (molecular function).

**Figure 3:**
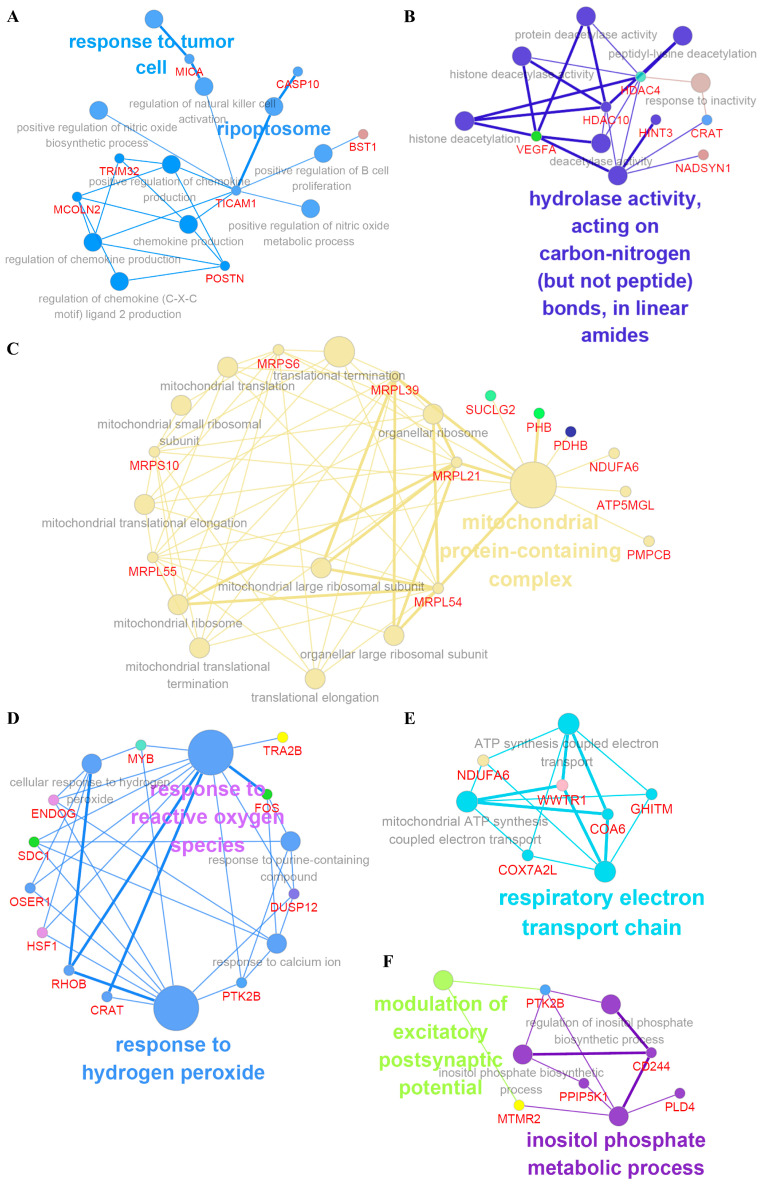
ClueGo analysis-based enrichment maps derived from GO terms associated with DEGs. Highly interconnected GO terms are presented. Terms in bold font indicate top GO terms. Gene names within subgroups were generated using ClueGO default settings. Biological processes shown include response to tumor cell (**A**), hydrolase activity (**B**), mitochondrial protein-containing complex (**C**), response to hydrogen peroxide (**D**), respiratory electron transport chain (**E**), and modulation of excitatory postsynaptic potential and inositol phosphate metabolic process (**F**). *FOS*, *ENDOG*, *MFPL21* and *COX7A2L* were identified as hub genes. All GO terms shown are statistically significant (*p* < 0.05 with Bonferroni correction).

Biological interpretation can be improved by constructing separate GO/pathway terminology networks using ClueGo and module analysis [14]. This analysis identified a number of additional processes associated with under-diagnoses, including interleukin-10 production (e.g., PHB, *CD40LG*, *SDC1*), synaptic vesicle transport (e.g., *BAG1*, *TOR1A*), mitochondrial protein containing complex (e.g., *PDHB*, *MRPS10*), and response to reactive oxygen species (e.g., *HSF1*, *RHOB*, *ENDOG*) (Figure 3A–F and Figure S1A–D, Tables S1 and S2). In addition, significant modules of protein–protein interaction networks were generated using MCODE and cytoHubba analysis with specific modules [15]. This subnetwork analysis identified *FOS* (fos proto-oncogene), *HIF1* (heat shock factor 1), *RHOB* (rho-related GTP-binding protein), *MRPS10* (mitochondrial ribosomal protein S10), *CAS9* (caspase 9), *IMP3* (U3 small nucleolar ribonucleoprotein), *HMMP* (hyaluronan mediated motility receptor), and *PPP2R4* (PP2A regulatory subunit B), among others, as hub genes (Figure S2A–J).

### 2.4. Validation of Candidates

**Figure 4:**
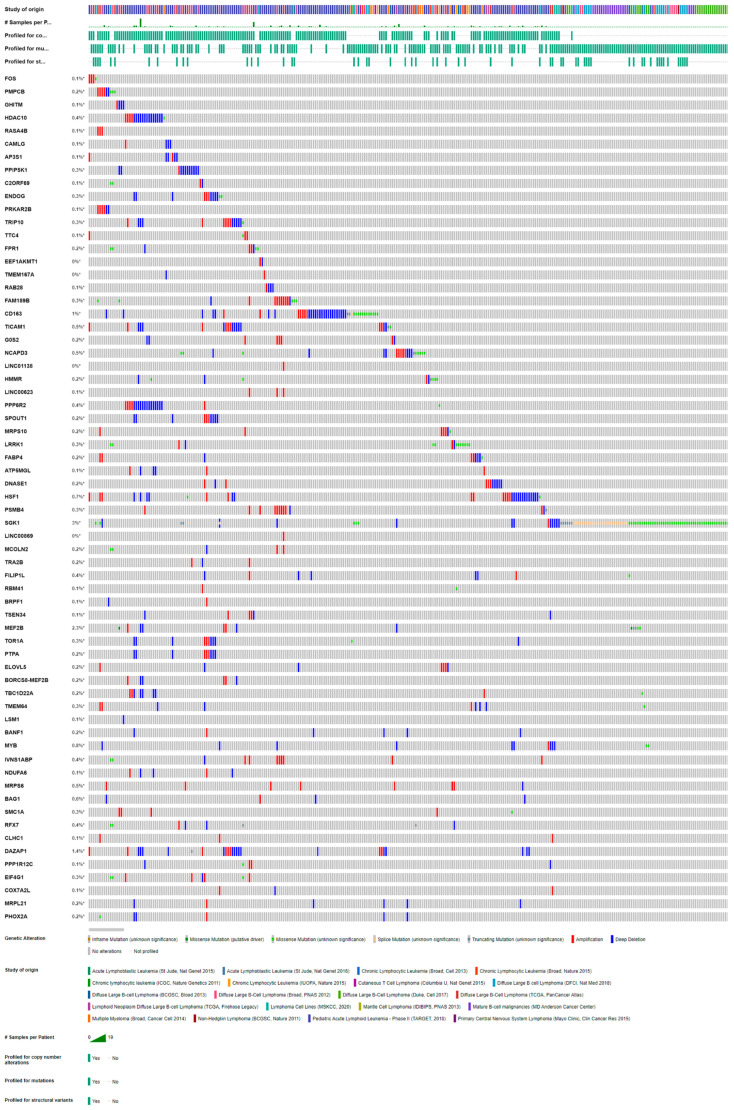
Verification of candidate genes by corporal analysis. Candidate genes were queried for genetic alterations in a cBioPortal dataset comprising 20 studies and 5905 patient samples (http://cbioportal.org/, accessed on 3 October 2022). Alterations were found in 0.3% to 3% of the respective analyses and are depicted graphically. Many candidate genes exhibited altered amplification. (* from 0.3% to 3%).

To select biomarker candidates for minimizing the under-diagnosis of FNAC, we selected the 65 genes with the greatest absolute log_2_ fold change. The molecular properties of the products of these gene are summarized in Table S3. We next verified gene expression profiles using cBioPortal for cancer genomics, which was originally developed for the interactive exploration of multidimensional cancer genomic datasets [16]. To this end, we extracted the dataset from the clinical presentation dataset of lymphoma containing 5905 patient samples from 20 different studies (Figure 4). Many of our candidates were either amplified or mutated in the extracted dataset, further strengthening the credibility of our candidate list.

**Figure 5:**
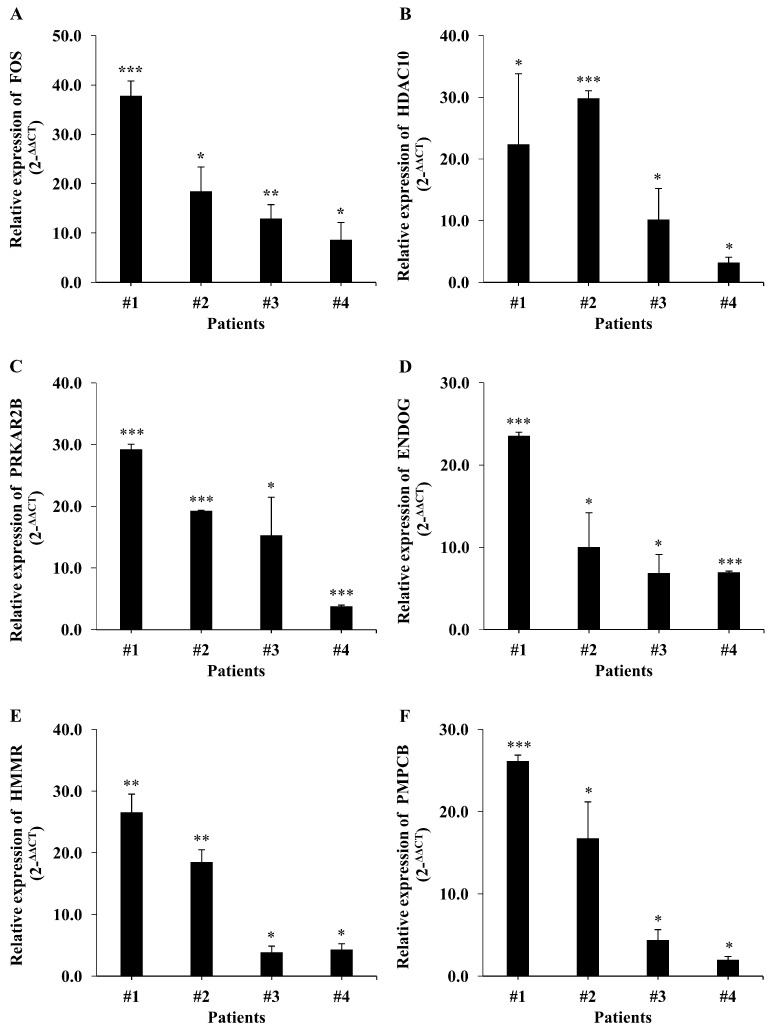
Verification of candidate genes by qRT-PCR analysis. (**A**–**F**) Graphic depiction of mRNA levels of the differentially expressed candidate genes FOS (**A**), *HDAC10* (**B**), *PRKAR2B* (**C**), *ENDOG* (**D**), *HMMR* (**E**) and *PMPCB* (**F**). All candidate genes were differentially expressed in NHL samples relative to benign samples. Data are expressed as means ± SD (*n* = 3; * *p* < 0.05, ** *p* < 0.01, *** *p* < 0.001).

After reviewing our analysis, we selected *FOS, HDAC10, PRKAR2B, ENDOG, HMMR* and *PMPCB*—the six genes with the highest fold changes—for validation. To this end, we performed a qRT-PCR analysis with the appropriate primers (Table S4), analyzing expression data normalized to the mean of *GAPDH* transcript levels using the 2^−ΔΔCT^ method. As shown in Figure 5A–F, these qRT-PCR analyses confirmed the upregulation of our candidate genes in NHL patients.

## 3. Discussion

Although Tru-cut biopsy could capture high-quality tissue samples, FNAC with Papanicolaou staining [17] is the first-line tool used in the evaluation of lymphadenopathy. The specimen obtained from FNAC could distinguish the majority of malignant tumors. However, there are some instances in which the diagnosis of a tumor remains obscure, notably including NHL. Therefore, samples processed using FNAC should be monitored for the presence of NHL. Reliable biomarkers capable of distinguishing NHL are crucial for accurate diagnosis of the malignancy. Molecular biomarkers not only improve our understanding of disease mechanisms in lymphomas, they also aid in classifying the outcomes of lymphoma patients. If we can correctly diagnose obscure cases during the FNAC step, we can minimize the risks associated with relying solely on FNAC in the diagnosis of lymphadenopathy.

In this paper, we proposed the use of *PMPCB* and *ENDOG*, encoding mitochondrial proteins, *PRKAR2B*, encoding a protein involved in energy production, and *CD168* (also known as HMMR), encoding a receptor involved cell motility and signaling, as biomarkers during the FNAC procedure for lymphadenopathy.

Our candidates include a number of proteins related to mitochondria, which are known to be involved in tumor formation and progression [18]. Mitochondria perform various fundamental functions including energy production, synthesis of macromolecules, redox modulation, and regulation of cell death [19]. Recently, Grasso et al. reported that oncogenic mitochondria are capable of transferring malignant capacities to recipient cells [20]. HMMR also drew our attention as an interesting candidate gene. Among the potential biological functions of HMMR are epithelial–mesenchymal transition (EMT) and G2M checkpoint pathways [21]. HMMR also participates in interferon-γ and interferon-α responses, all of which can promote carcinogenesis.

A recent report described an interesting idea involving the use of cytologic samples not only for diagnostic purposes but also for ancillary testing [22]. Yuan et al. applied this concept during the FNAC analysis of thyroid nodules, measuring the levels of *CCND2* and miR-206, identified as thyroid carcinoma biomarkers, directly by PCR after performing FNAC [23]. The process significantly reduced the number of procedures required to obtain an adequate biopsy sample while improving the accuracy of FNAC diagnosis. The use of rapid on-site biopsy to confirm the presence of tumors in a variety of malignancies is currently being evaluated in small-scale clinical trials [24]. Approaches that simultaneously analyze multiple biomarkers, such as microarrays or the newly developed slide-seq RNA analysis [25], could be key technologies for successful diagnosis. The slide-seq technique is a method for transferring RNA from a slide section onto a surface covered in nucleotide beads with fixed positions, enabling the location of the target RNA to be identified at a single-cell level. The growing number of predictive biomarkers heralds a potential paradigm shift in the care and handling of diagnostic samples.

With the discovery of novel biomarkers comes challenges in translating these markers into clinical practice [26]. The successful treatment of any disease requires the integration of diagnosis, prognosis, and evaluation of therapeutic targets. Collaborations among pathology, radiology and medicine are crucial in achieving these goals. In addition, studies of a larger populations are needed to verify the effectiveness of these markers in diagnosing patients with lymphadenopathy. Our hope is to make every effort to apply personalized medical insights to the treatment of patients.

## 4. Materials and Methods

### 4.1. Obtaining Specimens and Ancillary Tests

A total of 2517 cases of lymph node FNAC performed between January 2015 and December 2019 were retrospectively retrieved and evaluated. Diagnosis was confirmed by reviewing slides for cytology and histology using follow-up biopsy specimens from 432 patients. FNAC was performed by a radiologist in the Department of Radiology using Ultra-Sonography Guided Fine Needle Aspiration (USG-FNA) employing a 10 mL plastic syringe and standard 23 gauge needle without aspiration equipment. The collected material was smeared onto four glass slides and rapidly fixed in 95% ethyl alcohol. The sample remaining after rinsing the syringe with saline was used to make a cell block. FNAC smear slides were stained using an automated Papanicolaou Stainer (Thermo Scientific, Walldorf, Germany). Cell blocks were embedded in paraffin and sectioned at 4 μm thickness and stained with hematoxylin and eosin (H&E) using an automated H&E stainer (Dako CoverStainer, Glostrup, Denmark). For differential diagnosis, immunohistochemical (IHC) staining was performed on cell block slides using a Bond-III automated slide stainer (Leica Biosystems Melbourne Pty., Ltd., VIC, Melbourne, Australia).

### 4.2. RNA Isolation and RNA Sequencing

Remaining cell blocks from FNAC were used for RNA isolation. Total RNA was isolated using the QIAzol reagent (Qiagen, Hilden, Germany). RNA was quantified using a NanoDrop 2000 spectrophotometer (ND-2000; Thermo Fisher Scientific Inc., Waltham, DE, USA), and RNA quality (expressed as RNA integrity number) was assessed on an Agilent 2100 bioanalyzer using an RNA 6000 Nano Chip (Agilent Technologies, Amstelveen, Netherlands). RNA sequencing (RNA-seq) was performed on high-quality RNA samples (RNA integrity number > 7) from the six patient-derived organoids, RR1–3 and RS1–3. The six separate samples were multiplexed in each lane and sequenced on a HiSeq 4000 system (Illumina, San Diego, CA, USA). The sequenced libraries were aligned to the human genome (hg19) reference sequence using HISAT v2.1.0 [27]. The reference genome sequence and its annotation were downloaded from the UCSC genome browser (https://genome.ucsc.edu/, accessed on 10 October 2022).

### 4.3. Identification of Differentially Expressed Genes and Data Analysis

QuantSeq 3′ mRNA-Seq reads were aligned using Bowtie2 [28]. Bowtie2 indices were generated from the genome assembly sequence or the representative transcript sequences for alignment to the genome and transcriptome. The alignment file was used for assembling transcripts, estimating their abundances, and detecting differentially expressed genes (DEGs). DEGs were determined based on counts from unique and multiple alignments using coverage in Bedtools [29]. Read Count data were processed based on a quantile normalization method using edgeR, which is a Bioconductor software package (version 3.17) [30]. Data mining and graphic visualization were performed using Excel-based Differentially Expressed Gene Analysis (ExDEGA; Ebiogen Inc., Seoul, Republic of Korea). Probe sets without corresponding gene symbols were removed. In this study, differences with a *p*-value < 0.05 and absolute log_2_ (fold change) ≥ 1 were considered statistically significant.

### 4.4. Gene Set Enrichment Analysis (GSEA)

Gene set enrichment analysis (GSEA), a computational technique that examines the statistical significance and consistency of differences between two biological states in a set of genes that has been defined in advance (e.g., phenotypes), was performed as described previously [31].

### 4.5. Heatmap with MeV

Clustering analyses were performed in MultiExperiment Viewer (MeV), a Java-based application that enables the advanced analysis of gene expression data through an intuitive graphical user interface, as described by the producers of MeV (The Institute for Genomic Research, Rockville, MD, USA).

### 4.6. Gene Ontology with DAVID

The roles of DEGs, subdivided into the categories molecular function (MF), biological process (BP) and cellular component (CC), were determined by gene ontology (GO) analysis using DAVID (Database for Annotation, Visualization and Integrated Discovery; https://david.ncifcrf.gov/, accessed on 15 September 2022), which is an online biological information database. A *p*-value < 0.05 constituted the cut-off criterion.

### 4.7. Significant Module Analysis with Cytoscape

Protein–protein interaction (PPI) networks were mapped using Cytoscape (version 3.9.1; https://cytoscape.org/, accessed on 15 September 2022), which is a public-access software that can graphically edit, display, and analyze the network. Significant modules in PPI networks were identified using Molecular Complex Detection (MCODE), a plug-in app of Cytoscape designed to analyze densely connected regions by clustering a given network. Hub genes were identified using the cytoHubba analysis in Cytoscape.

### 4.8. ClueGO

ClueGO, a Cytoscape plugin that facilitates data visualization for biological interpretations of large lists of genes, is useful for analyzing gene or protein interaction networks through visualization. Biological information was captured using GO categories. The network specificity function of ClueGO displays GO terms according to levels, with low-level settings displaying general terms and higher levels displaying highly specific terms. Each GO is shown as a node connected to edges, which indicate interactions. The enrichment significance of terms is reflected in the size of the corresponding nodes. The network is automatically placed using the organic layout algorithm supported by Cytoscape software version 3.9.1.

### 4.9. Validation of Genetic Alterations in Candidate Genes

Genetic alterations in candidate genes in the lympoma adenocarcinoma dataset were analyzed using cBioPortal (http://cbioportal.org/, accessed on 3 October 2022), which is an online analysis platform for multidimensional cancer genomic data that provides the collective visualization of genes, samples, and data types.

### 4.10. Reverse Transcription-Polymerase Chain Reaction (RT-PCR) and Quantitative RT-PCR (qRT-PCR)

Total RNA was extracted using the TRIzol reagent (Invitrogen, Carlsbad, CA, USA) according to the manufacturer’s instructions. RNA purity and concentration were determined by spectrophotometry. Reverse transcription was carried out using an amfiRivert cDNA Synthesis Platinum Master Mix (GenDEPOT, Baker, TX, USA; cat. no. R5600-500) according to the manufacturer’s instructions. For qRT-PCR, the desired cDNA fragments were amplified using a Mic qPCR Cycler for real-time PCR (Bio Molecular Systems, Coomera, Australia) in reaction mixes (total volume, 30 μL) containing 1 μL cDNA, 5X Hot FIREPol EvaGreen qPCR Supermix (Solis Bio Dyne, Tartu, Estonia) and the relevant primers (see Table S4). The cycling conditions used were 95 °C for 60 s, which was followed by 40 cycles of denaturation at 95 °C for 15 s and annealing/extension at 60 °C for 30 s. Data were recorded as the cycle threshold (Ct) on a Mic Real-Time PCR system using analytical software (Bio Molecular Systems, version 2.10.4) from the same manufacturer. mRNA levels for the gene of interest were quantified using the 2^−ΔΔCq^ method and normalized to those of the housekeeping gene, *GAPDH* (glyceraldehyde-3-phosphate dehydrogenase).

### 4.11. Statistical Analysis

All experiments were performed in at least triplicate (*n* ≥ 3), and data are presented as means ± standard deviation (SD). The significance of differences between two experimental means was determined with Student’s *t* test for independent samples using SPSS software version 26.0. *p*-values < 0.05 were considered statistically significant.

## Figures and Tables

**Table 1:** Clinical characteristics of the patients.

Sample No.	Case 1	Case 2	Case 3	Case 4	Case 5	Case 6
Sex	F	M	F	F	M	F
Age (years)	67	59	78	76	59	64
History	Palpable, painless	TB	HL, Colon Ca.	NSL	NSL	Rectal Ca.
Duration						
<4 weeks						
≥4 weeks	o	o	o	o	o	o
Tumor length						
<1 cm		o	o		o	o
>1 cm	o			o		
Number of enlarged LN						
1						o
2 or more	o	o	o	o	o	
Location	Axillary	Cervical	Axillary	Cervical	Submandibular	Inguinal
Pathological Diagnosis	DLBCL	MCL	DLBCL	FL	RH	RH

Abbreviation: F, Female; M, Male; TB, Tuberculosis; HL, Hodgkin lymphoma; ca., carcinoma; LN, Lymph node; DLBCL, Diffuse large B cell lymphoma; MCL, Mantle cell lymphoma; FL, Follicular lymphoma; RH, Reactive hyperplasia.

**Table 2:** Gene set enrichment analysis of non-Hodgkin lymphoma-related DEGs.

Gene Sets Details	SIZE	ES	NES	NOM *p*-Value	FDR *q*-Value
KEGG_METABOLISM_OF_XENOBIOTICS_BY_CYTOCHROME_P450	70	0.696	2.05	0.000	0.000
KEGG_DRUG_METABOLISM_CYTOCHROME_P450	72	0.669	2.01	0.000	0.001
KEGG_PEROXISOME	78	0.602	1.82	0.000	0.005
KEGG_PROXIMAL_TUBULE_BICARBONATE_RECLAMATION	23	0.751	1.81	0.000	0.005
KEGG_PPAR_SIGNALING_PATHWAY	69	0.613	1.81	0.000	0.004
KEGG_GLUTATHIONE_METABOLISM	50	0.635	1.81	0.000	0.004
KEGG_PENTOSE_AND_GLUCURONATE_INTERCONVERSIONS	28	0.704	1.77	0.000	0.006
KEGG_ASCORBATE_AND_ALDARATE_METABOLISM	25	0.722	1.75	0.002	0.009
KEGG_ARACHIDONIC_ACID_METABOLISM	58	0.595	1.74	0.000	0.010
KEGG_RETINOL_METABOLISM	64	0.595	1.73	0.002	0.011
KEGG_DRUG_METABOLISM_OTHER_ENZYMES	51	0.610	1.72	0.000	0.011
KEGG_STEROID_HORMONE_BIOSYNTHESIS	55	0.594	1.70	0.002	0.016
KEGG_PORPHYRIN_AND_CHLOROPHYLL_METABOLISM	41	0.626	1.69	0.004	0.015
KEGG_GLYCOLYSIS_GLUCONEOGENESIS	62	0.582	1.69	0.002	0.015
KEGG_PYRUVATE_METABOLISM	40	0.613	1.65	0.008	0.022
KEGG_ARGININE_AND_PROLINE_METABOLISM	54	0.584	1.65	0.004	0.021
KEGG_SELENOAMINO_ACID_METABOLISM	26	0.661	1.61	0.008	0.030
KEGG_ALANINE_ASPARTATE_AND_GLUTAMATE_METABOLISM	32	0.629	1.60	0.011	0.035
KEGG_STARCH_AND_SUCROSE_METABOLISM	52	0.552	1.57	0.009	0.044

Abbreviations: ES, enrichment score; NES, normalized enrichment score; NOM *p*-value, normalized *p*-value; FDR, false discovery rate.

**Table 3:** Gene ontology (GO) analysis of NHL-related DEGs.

Category	Term	Description	Count	*p*-Value
**BP**	GO:0007229	Integrin-mediated signaling pathway	7	1.9 × 10^−3^
	GO:0032543	Mitochondrial translation	6	5.6 × 10^−3^
	GO:0042493	Response to drug	10	8.2 × 10^−3^
	GO:0043129	Surfactant homeostasis	3	1.5 × 10^−2^
	GO:0006355	Regulation of transcription, DNA-templated	20	2.4 × 10^−2^
	GO:1990830	Cellular response to leukemia inhibitory factor	5	3.3 × 10^−2^
	GO:0007155	Cell adhesion	13	3.3 × 10^−2^
	GO:0048026	Positive regulation of mRNA splicing, via spliceosome	3	3.7 × 10^−2^
	GO:0007160	Cell-matrix adhesion	5	3.8 × 10^−2^
	GO:0001570	Vasculogenesis	4	3.8 × 10^−2^
	GO:0006886	Intracellular protein transport	9	4.1 × 10^−2^
	GO:0021773	Striatal medium spiny neuron differentiation	2	4.6 × 10^−2^
	GO:0060271	Cilium assembly	7	4.8 × 10^−2^
	GO:0048010	Vascular endothelial growth factor receptor signaling pathway	3	4.8 × 10^−2^
**CC**	GO:0005739	Mitochondrion	34	1.5 × 10^−4^
	GO:0005829	Cytosol	86	1.6 × 10^−3^
	GO:0009986	Cell surface	17	3.5 × 10^−3^
	GO:0005856	Cytoskeleton	15	4.7 × 10^−3^
	GO:0005737	Cytoplasm	83	9.1 × 10^−3^
	GO:0005769	Early endosome	10	1.1 × 10^−2^
	GO:0005743	Mitochondrial inner membrane	12	2.1 × 10^−2^
	GO:0043025	Neuronal cell body	11	2.2 × 10^−2^
	GO:0034686	Integrin alphav-beta8 complex	2	2.3 × 10^−2^
	GO:0016021	Integral component of membrane	78	2.6 × 10^−2^
	GO:0005762	Mitochondrial large ribosomal subunit	4	2.9 × 10^−2^
	GO:0005790	Smooth endoplasmic reticulum	3	3.9 × 10^−2^
**MF**	GO:0004725	Protein tyrosine phosphatase activity	6	9.1 × 10^−3^
	GO:0008138	Protein tyrosine/serine/threonine phosphatase activity	4	1.0 × 10^−2^
	GO:0005515	Protein binding	163	4.0 × 10^−2^
	GO:0016787	Hydrolase activity	8	4.5 × 10^−2^
	GO:0050839	Cell adhesion molecule binding	4	5.1 × 10^−2^

## Data Availability

Data sharing not applicable.

## Supplementary Materials

The supporting information can be downloaded at: https://www.mdpi.com/article/10.3390/ijms241512394/s1.
