# Supplementary material for: Analysis of Under-Diagnosed Malignancy during Fine Needle Aspiration Cytology of Lymphadenopathies
Source: Int J Mol Sci. 2023 Aug 3;24(15):12394. doi: 10.3390/ijms241512394 (PMC10418811; doi:10.3390/ijms241512394)
Supplement: Supplementary file 1 [file ijms-24-12394-s001.zip › Supplementary figure.pptx]

## Slide 1
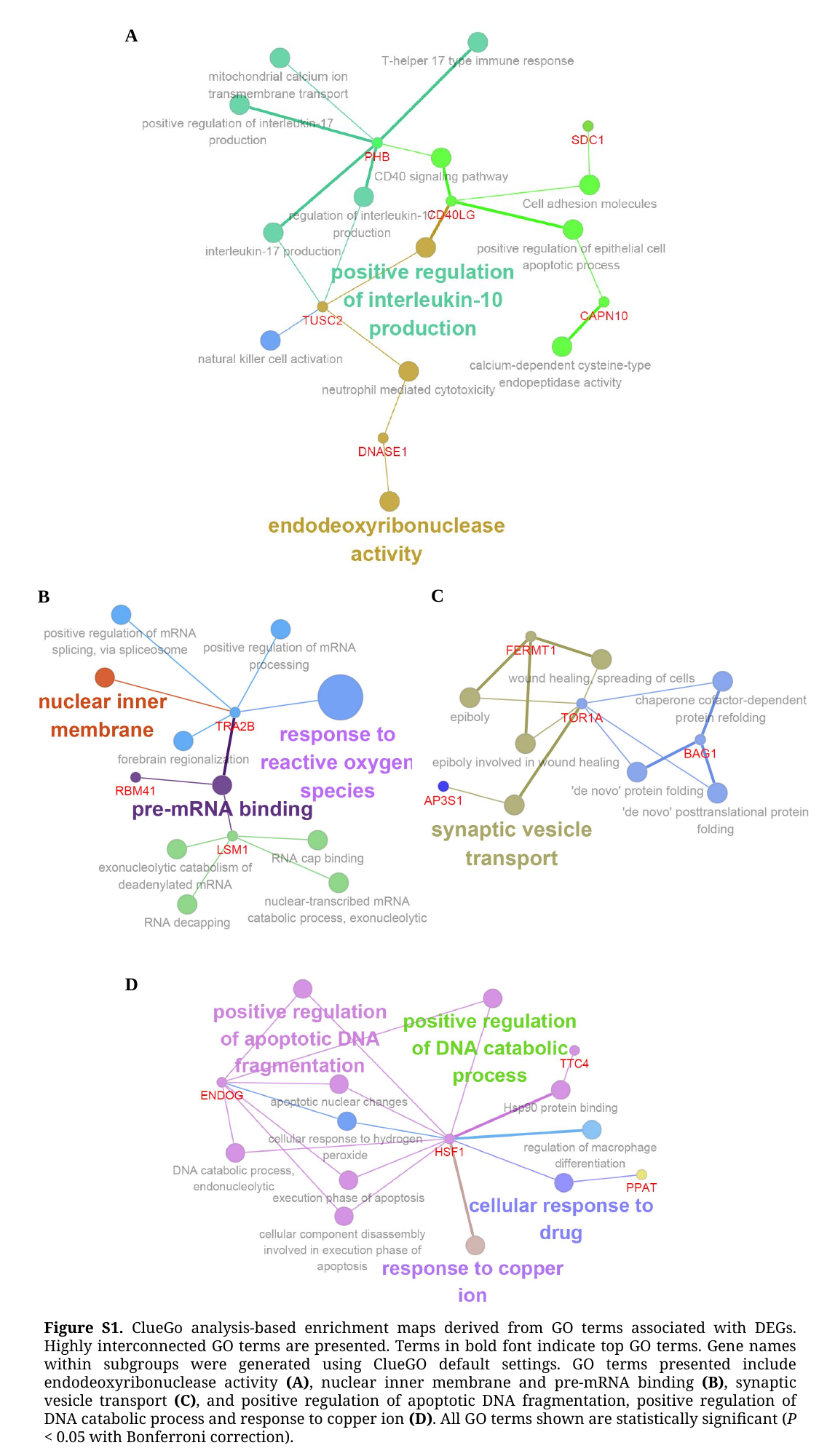

A
C
B
D
Figure S1. ClueGo analysis-based enrichment maps derived from GO terms associated with DEGs. Highly interconnected GO terms are presented. Terms in bold font indicate top GO terms. Gene names within subgroups were generated using ClueGO default settings. GO terms presented include endodeoxyribonuclease activity (A), nuclear inner membrane and pre-mRNA binding (B), synaptic vesicle transport (C), and positive regulation of apoptotic DNA fragmentation, positive regulation of DNA catabolic process and response to copper ion (D). All GO terms shown are statistically significant (P < 0.05 with Bonferroni correction).

## Slide 2
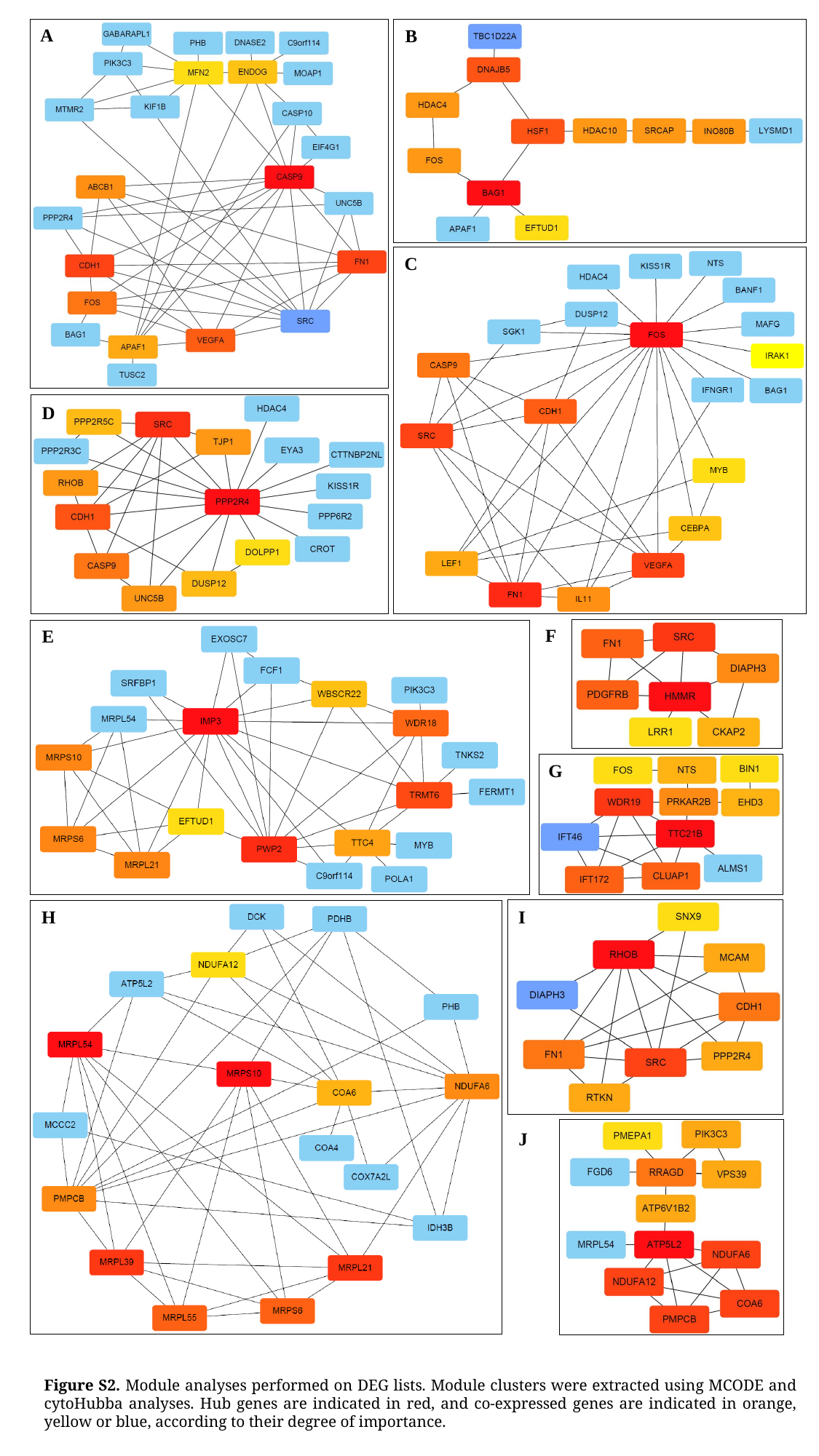

A
B
C
D
F
E
G
H
I
J
Figure S2. Module analyses performed on DEG lists. Module clusters were extracted using MCODE and cytoHubba analyses. Hub genes are indicated in red, and co-expressed genes are indicated in orange, yellow or blue, according to their degree of importance.
