# Supplementary material for: Analysis of Under-Diagnosed Malignancy during Fine Needle Aspiration Cytology of Lymphadenopathies
Source: Int J Mol Sci. 2023 Aug 3;24(15):12394. doi: 10.3390/ijms241512394 (PMC10418811; doi:10.3390/ijms241512394)
Supplement: Supplementary file 1 [file ijms-24-12394-s001.zip › Table S1. 94 up-regulated genes.pdf]

Table S1. 94 up-regulated genes.

	Genes	Identifier	Description
1	FOS	NM_005252	Fos proto-oncogene, AP-1 trancription factor subunit
2	CLNS1A	NM_001293	chloride nucleotide-sensitive channel 1A
3	PMPCB	NM_004279	peptidase, mitochondrial processing beta subunit
4	GHITM	NM_014394	growth hormone inducible transmembrane protein
5	HDAC10	NM_032019	histone deacetylase 10
6	RASA4B	NM_001277335	RAS p21 protein activator 4B
7	PPAT	NM_002703	phosphoribosyl pyrophosphate amidotransferase
8	OSBPL10	NM_001174060	oxysterol binding protein like 10
9	CAMLG	NM_001745	calcium modulating ligand
10	RHOB	NM_004040	ras homolog family member B
11	CKAP2	NM_001098525	cytoskeleton associated protein 2
12	TUSC2	NM_007275	tumor suppressor candidate 2
13	GLUD1P3	NR_048575	glutamate dehydrogenase 1 pseudogene 3
14	HPS5	NM_007216	HPS5, biogenesis of lysosomal organelles complex 2 subunit 2
15	AP3S1	NM_001284	adaptor related protein complex 3 sigma 1 subunit
16	PPIP5K1	NM_014659	diphosphoinositol pentakisphosphate kinase 1
17	C2orf69	NM_153689	chromosome 2 open reading frame 69
18	OSER1	NM_016470	oxidative stress responsive serine rich 1
19	DRAM1	NM_018370	DNA damage regulated autophagy modulator 1
20	ENDOG	NM_004435	endonuclease G
21	PRKAR2B	NM_002736	protein kinase cAMP-dependent type II regulatory subunit beta
22	FAM159A	NM_001042693	family with sequence similarity 159 member A
23	TRIP10	NR_110231	thyroid hormone receptor interactor 10
24	TTC4	NM_004623	tetratricopeptide repeat domain 4
25	FPR1	NM_001193306	formyl peptide receptor 1
26	SAAL1	NM_138421	serum amyloid A like 1
27	N6AMT2	NM_174928	EEF1AKMT1, EEF1A Lysine Methyltransferase 1
28	TMEM167A	NM_174909	transmembrane protein 167A
29	RAB28	NM_001159601	RAB28, member RAS oncogene family
30	ZNF664	NM_001204298	zinc finger protein 664
31	FAM189B	NM_198264	family with sequence similarity 189 member B

32	SUCLG2	NM_001177599	succinate-CoA ligase GDP-forming beta subunit
33	LOC155060	NR_036573	AI894139 pseudogene
34	CD163	NM_203416	CD163 molecule
35	TICAM1	NM_182919	toll like receptor adaptor molecule 1
36	G0S2	NM_015714	G0 /G1 switch 2
37	NCAPD3	NM_015261	non-SMC condensin II complex subunit D3
38	LINC01138	NR_104014	long intergenic non-protein coding RNA 1138
39	HMMR	NM_001142556	hyaluronan mediated motility receptor
40	DCK	NM_000788	deoxycytidine kinase
41	LINC00623	NR_024511	long intergenic non-protein coding RNA 623
42	PPP6R2	NM_001242900	protein phosphatase 6 regulatory subunit 2
43	C9orf114	NM_016390	chromosome 9 open reading frame 114
44	MRPS10	NM_018141	mitochondrial ribosomal protein S10
45	LINC-PINT	NR_110473	long intergenic non-protein coding RNA, p53 induced transcript
46	LOC374443	NR_046450	C-type lectin domain family 2 member D pseudogene
47	RP9	NM_203288	retinitis pigmentosa 9 (autosomal dominant)
48	LRRK1	NM_024652	leucine-rich repeat kinase 1
49	FABP4	NM_001442	fatty acid binding protein 4
50	ZDHHC16	NM_032327	zinc finger DHHC-type containing 16
51	ATP5L2	NM_001165877	ATP synthase, H+ transporting, mitochondrial Fo complex subunit G2
52	DNASE1	NM_005223	deoxyribonuclease I
53	HSF1	NM_005526	heat shock transcription factor 1
54	ICE2	NM_001018089	interactor of little elongation complex ELL subunit 2
55	PHB	NM_001281497	prohibitin
56	PSMB4	NM_002796	proteasome subunit beta 4
57	SGK1	NM_001291995	serum /glucocorticoid regulated kinase 1
58	LINC00869	NR_111953	long intergenic non-protein coding RNA 869
59	MCOLN2	NM_153259	mucolipin 2
60	LINC00106	NR_130733	long intergenic non-protein coding RNA 106
61	ATP6V1B2	NM_001693	ATPase H+ transporting V1 subunit B2
62	TRA2B	NM_004593	transformer 2 beta homolog (Drosophila)
63	FILIP1L	NM_001282794	filamin A interacting protein 1-like
64	EYA3	NM_001282561	EYA transcriptional coactivator and phosphatase 3

65	RBM41	NM_001171080	RNA binding motif protein 41
66	BRPF1	NM_004634	bromodomain and PHD finger containing 1
67	TSEN34	NM_001282333	tRNA splicing endonuclease subunit 34
68	MEF2B	NM_001145785	myocyte enhancer factor 2B
69	TOR1A	NM_000113	torsin family 1 member A
70	PPP2R4	NM_021131	protein phosphatase 2A regulatory subunit 4
71	ZSWIM6	NM_020928	zinc finger SWIM-type containing 6
72	ELOVL5	NM_021814	ELOVL fatty acid elongase 5
73	MEF2BNB-MEF2B	NR_027307	BORCS8-MEF2B, transcript variant 2, non-coding RNA
74	TBC1D22A	NM_001284303	TBC1 domain family member 22A
75	COA4	NM_016565	cytochrome c oxidase assembly factor 4 homolog
76	TMEM64	NM_001008495	transmembrane protein 64
77	LSM1	NR_045492	LSM1 homolog, mRNA degradation associated
78	BANF1	NM_001143985	barrier to autointegration factor 1
79	MYB	NM_005375	MYB proto-oncogene, transcription factor
80	IVNS1ABP	NM_006469	influenza virus NS1A binding protein
81	NDUFA6	NM_002490	NADH:ubiquinone oxidoreductase subunit A6
82	MRPS6	NM_032476	mitochondrial ribosomal protein S6
83	BAG1	NM_001172415	BCL2 associated athanogene 1
84	SMC1A	NM_001281463	structural maintenance of chromosomes 1A
85	RFX7	NM_022841	regulatory factor X7
86	PTK2B	NM_173174	protein tyrosine kinase 2 beta
87	FBXL15	NM_024326	F-box and leucine-rich repeat protein 15
88	CLHC1	NM_152385	clathrin heavy chain linker domain containing 1
89	DAZAP1	NM_170711	DAZ associated protein 1
90	PPP1R12C	NM_017607	protein phosphatase 1 regulatory subunit 12C
91	EIF4G1	NM_198241	eukaryotic translation initiation factor 4 gamma 1
92	COX7A2L	NM_004718	cytochrome c oxidase subunit 7A2 like
93	MRPL21	NM_181514	mitochondrial ribosomal protein L21
94	PHOX2A	NM_005169	paired like homeobox 2a
