# Supplementary material for: Analysis of Under-Diagnosed Malignancy during Fine Needle Aspiration Cytology of Lymphadenopathies
Source: Int J Mol Sci. 2023 Aug 3;24(15):12394. doi: 10.3390/ijms241512394 (PMC10418811; doi:10.3390/ijms241512394)
Supplement: Supplementary file 1 [file ijms-24-12394-s001.zip › Table S2. 194 down-regulated genes.pdf]

Table S2. 194 down-regulated genes.

	Genes	Identifier	Description
1	FOCAD	NM_017794	focadhesin
2	SDC1	NM_001006946	syndecan 1
3	BRF2	NM_018310	BRF2, RNA polymerase III transcription initiation factor 50 kDa subunit
4	ANKRD36BP2	NR_015424	ankyrin repeat domain 36B pseudogene 2
5	LOC100289561	NM_001242713	uncharacterized LOC100289561
6	GBA2	NM_020944	glucosylceramidase beta 2
7	HSDL2	NM_032303	hydroxysteroid dehydrogenase like 2
8	WWTR1	NM_001168280	WW domain containing transcription regulator 1
9	CASP10	NM_001306083	caspase 10
10	MRPL39	NM_017446	mitochondrial ribosomal protein L39
11	MXD1	NM_001202513	MAX dimerization protein 1
12	FGF12	NM_021032	fibroblast growth factor 12
13	ZNF507	NM_014910	zinc finger protein 507
14	MYO1B	NM_001130158	myosin IB
15	DGCR2	NM_001184781	DiGeorge syndrome critical region gene 2
16	HID1	NM_030630	HID1 domain containing
17	THRA1 /BTR	NR_131239	uncharacterized LOC105371807
18	GPATCH1	NM_018025	G-patch domain containing 1
19	BET1	NM_005868	Bet1 golgi vesicular membrane trafficking protein
20	SRI	NM_198901	sorcin
21	PTP4A3	NM_007079	protein tyrosine phosphatase type IVA, member 3
22	RPL19P12	NR_026660	ribosomal protein L19 pseudogene 12
23	CRAT	NM_001257363	carnitine O-acetyltransferase
24	DUSP12	NM_007240	dual specificity phosphatase 12
25	HINT3	NM_138571	histidine triad nucleotide binding protein 3
26	ZNF407	NM_001146189	zinc finger protein 407
27	RBMS3	NM_001003792	RNA binding motif single stranded interacting protein 3
28	CLDND1	NM_001040199	claudin domain containing 1
29	KEL	NM_000420	Kell blood group, metallo-endopeptidase
30	TMEM42	NM_144638	transmembrane protein 42
31	LHFP	NM_005780	lipoma HMGIC fusion partner
32	HS3ST3B1	NR_130138	heparan sulfate-glucosamine 3-sulfotransferase 3B1
33	COLCA2	NM_001271458	colorectal cancer associated 2
34	SLC16A3	NM_001042422	solute carrier family 16 member 3
35	PIK3R3	NM_003629	phosphoinositide-3-kinase regulatory subunit 3
36	JMJD4	NM_001161465	jumonji domain containing 4
37	GNLY	NM_001302758	granulysin
38	KCNG1	NM_002237	potassium voltage-gated channel modifier subfamily G member 1
39	NR2F1-AS1	NR_109825	NR2F1 antisense RNA 1
40	PLD4	NM_138790	phospholipase D family member 4
41	INHBA	NM_002192	inhibin beta A
42	ABCD4	NR_003256	ATP binding cassette subfamily D member 4
43	PFN2	NM_002628	profilin 2
44	LY9	NM_002348	lymphocyte antigen 9
45	TSPAN5	NM_005723	tetraspanin 5

46	VEGFA	NM_001171625	vascular endothelial growth factor A
47	DEXI	NM_014015	Dexi homolog
48	KISS1R	NM_032551	KISS1 receptor
49	EXOSC7	NM_015004	exosome component 7
50	LOC101927730	NR_110841	uncharacterized LOC101927730
51	KDM3A	NM_018433	lysine demethylase 3A
52	NADSYN1	NM_018161	NAD synthetase 1
53	GSTM4	NR_024538	glutathione S-transferase mu 4
54	BACE1	NM_012104	beta-site APP-cleaving enzyme 1
55	EDNRB-AS1	NR_103853	EDNRB antisense RNA 1
56	DCAF17	NR_028482	DDB1 and CUL4 associated factor 17
57	NUP35	NM_001287584	nucleoporin 35kDa
58	ZNRF2	NM_147128	zinc and ring finger 2, E3 ubiquitin protein ligase
59	KIF16B	NM_024704	kinesin family member 16B
60	PDHB	NR_033384	pyruvate dehydrogenase (lipoamide) beta
61	PTPRF	NM_002840	protein tyrosine phosphatase, receptor type F
62	ADAM22	NM_021721	ADAM metallopeptidase domain 22
63	CDR1	NM_004065	cerebellar degeneration related protein 1
64	CAPN10	NM_023085	calpain 10
65	RAB20	NM_017817	RAB20, member RAS oncogene family
66	LINC01530	NR_034159	long intergenic non-protein coding RNA 1530
67	CADM3-AS1	NR_037870	CADM3 antisense RNA 1
68	MARK1	NM_018650	microtubule affinity regulating kinase 1
69	WLS	NM_001002292	wntless Wnt ligand secretion mediator
70	STRADB	NM_001206864	STE20-related kinase adaptor beta
71	C1RL-AS1	NR_026947	C1RL antisense RNA 1
72	WDR19	NM_025132	WD repeat domain 19
73	MMEL1	NM_033467	membrane metallo-endopeptidase-like 1
74	LOC100506136	NR_038948	uncharacterized LOC100506136
75	RTKN	NM_001015056	rhotekin
76	F5	NM_000130	coagulation factor V
77	ZSCAN25	NM_145115	zinc finger and SCAN domain containing 25
78	ZNF625-ZNF20	NR_037802	ZNF625-ZNF20 readthrough (NMD candidate)
79	ZNF20	NM_021143	zinc finger protein 20
80	B3GAT1	NM_018644	beta-1,3-glucuronyltransferase 1
81	ISLR	NM_005545	immunoglobulin superfamily containing leucine-rich repeat
82	LRP3	NM_002333	LDL receptor related protein 3
83	ABCA3	NM_001089	ATP binding cassette subfamily A member 3
84	SNHG17	NR_027241	small nucleolar RNA host gene 17
85	XKR8	NM_018053	XK related 8
86	PCNXL4	NM_022495	.
87	DPY19L1	NM_015283	dpy-19 like 1 (C. elegans)
88	TTC21B	NM_024753	tetratricopeptide repeat domain 21B
89	ATXN7L2	NM_153340	ataxin 7 like 2
90	DUSP7	NM_001947	dual specificity phosphatase 7
91	MAB21L2	NM_006439	mab-21 like 2
92	TRIM32	NM_012210	tripartite motif containing 32
93	LACTB	NM_171846	lactamase beta

94	SRGAP2D	NR_120535_1	SLIT-ROBO Rho GTPase activating protein 2D (pseudogene)
95	SULF1	NM_015170	sulfatase 1
96	PNMA3	NM_013364	paraneoplastic Ma antigen 3
97	RYR3	NM_001243996	ryanodine receptor 3
98	TOR1AIP1	NM_015602	torsin 1A interacting protein 1
99	UBTD1	NM_024954	ubiquitin domain containing 1
100	PIGY	NM_001042616	phosphatidylinositol glycan anchor biosynthesis class Y
101	PYURF	NM_032906	PIGY upstream reading frame
102	FERMT1	NM_017671	fermitin family member 1
103	MPPED2	NM_001145399	metallophosphoesterase domain containing 2
104	LOC101928517	NR_110732	uncharacterized LOC101928517
105	NIFK-AS1	NR_037858	NIFK antisense RNA 1
106	ZNF75D	NR_110381	zinc finger protein 75D
107	TSPAN32	NM_139022	tetraspanin 32
108	RAB3IL1	NM_001271686	RAB3A interacting protein like 1
109	NID1	NM_002508	nidogen 1
110	SIGLEC5	NM_003830	sialic acid binding Ig like lectin 5
111	SLC35B4	NM_032826	solute carrier family 35 member B4
112	TMEM209	NM_001301163	transmembrane protein 209
113	LRRC19	NM_022901	leucine rich repeat containing 19
114	INO80B	NM_031288	INO80 complex subunit B
115	NUDT18	NM_024815	nudix hydrolase 18
116	LINC00667	NR_015389	long intergenic non-protein coding RNA 667
117	MPND	NM_001300862	MPN domain containing
118	CD40LG	NM_000074	CD40 ligand
119	DDX43	NM_018665	DEAD-box helicase 43
120	ZNF549	NM_153263	zinc finger protein 549
121	ZKSCAN3	NM_024493	zinc finger with KRAB and SCAN domains 3
122	HDAC4	NM_006037	histone deacetylase 4
123	LZTFL1	NM_001276379	leucine zipper transcription factor like 1
124	DHODH	NM_001361	dihydroorotate dehydrogenase (quinone)
125	SERINC5	NR_126060	serine incorporator 5
126	TCL6	NR_028288	T-cell leukemia /lymphoma 6 (non-protein coding)
127	NUDT12	NM_001300741	nudix hydrolase 12
128	FAM210A	NM_152352	family with sequence similarity 210 member A
129	MRPL54	NM_172251	mitochondrial ribosomal protein L54
130	LINC01296	NR_122112	long intergenic non-protein coding RNA 1296
131	SRCAP	NM_006662	Snf2-related CREBBP activator protein
132	L1CAM	NM_000425	L1 cell adhesion molecule
133	KIF13A	NM_001105568	kinesin family member 13A
134	SMCO4	NM_020179	single-pass membrane protein with coiled-coil domains 4
135	ENOSF1	NM_001126123	enolase superfamily member 1
136	CAPN11	NM_007058	calpain 11
137	FGD1	NM_004463	FYVE, RhoGEF and PH domain containing 1
138	TVP23C-CDRT4	NR_037924	TVP23C-CDRT4 readthrough
139	MICA	NM_001289153	MHC class I polypeptide-related sequence A
140	TMEM38B	NM_018112	transmembrane protein 38B

141	ITGB8	NM_002214	integrin subunit beta 8
142	RFX2	NM_134433	regulatory factor X2
143	GPR182	NM_007264	G protein-coupled receptor 182
144	HOXA5	NM_019102	homeobox A5
145	GNA14	NM_004297	G protein subunit alpha 14
146	KRT72	NM_001146225	keratin 72
147	EHD3	NM_014600	EH domain containing 3
148	IL11	NM_000641	interleukin 11
149	ADGRF5	NM_001098518	adhesion G protein-coupled receptor F5
150	ZNF577	NR_024181	zinc finger protein 577
151	ZNF747	NM_001305019	zinc finger protein 747
152	RAD54B	NM_012415	RAD54 homolog B (S. cerevisiae)
153	TMEM159	NR_125971	transmembrane protein 159
154	POSTN	NM_001135936	periostin
155	COA6	NM_001206641	cytochrome c oxidase assembly factor 6
156	PARG	NR_130169	poly(ADP-ribose) glycohydrolase
157	C10orf32	NM_144591	.
158	COMTD1	NM_144589	catechol-O-methyltransferase domain containing 1
159	MTMR2	NM_201278	myotubularin related protein 2
160	CRB2	NM_173689	crumbs 2, cell polarity complex component
161	CARKD	NM_001242883	.
162	CHPF	NM_001195731	chondroitin polymerizing factor
163	TMEM135	NR_033149	transmembrane protein 135
164	CDH1	NM_004360	cadherin 1
165	EXOC3L4	NM_001077594	exocyst complex component 3 like 4
166	SEZ6L2	NM_012410	seizure related 6 homolog like 2
167	BST1	NM_004334	bone marrow stromal cell antigen 1
168	MYO6	NM_001300899	myosin VI
169	ARSB	NM_000046	arylsulfatase B
170	ABCB1	NM_000927	ATP-binding cassette, sub-family B (MDR /TAP), member 1
171	CD244	NM_001166664	CD244 molecule
172	ADRA2A	NM_000681	adrenoceptor alpha 2A
173	SCN9A	NM_002977	sodium voltage-gated channel alpha subunit 9
174	CBR1	NM_001757	carbonyl reductase 1
175	PDE8B	NM_003719	phosphodiesterase 8B
176	DIAPH3	NM_001258367	diaphanous related formin 3
177	PWP2	NM_005049	PWP2 periodic tryptophan protein homolog (yeast)
178	UCHL1	NM_004181	ubiquitin C-terminal hydrolase L1
179	PSD3	NM_015310	pleckstrin and Sec7 domain containing 3
180	FAM161A	NR_037710	family with sequence similarity 161 member A
181	WDR91	NM_014149	WD repeat domain 91
182	NPR2	NM_003995	natriuretic peptide receptor 2
183	PIK3CD-AS2	NR_126366	PIK3CD antisense RNA 2
184	ITGAV	NM_001145000	integrin subunit alpha V
185	CELSR2	NM_001408	cadherin EGF LAG seven-pass G-type receptor 2
186	RBAK	NM_001204456	RB associated KRAB zinc finger
187	MRPL55	NM_181462	mitochondrial ribosomal protein L55
188	MFAP3	NM_005927	microfibrillar associated protein 3
189	LRR1	NM_152329	leucine rich repeat protein 1

190	TSPAN7	NM_004615	tetraspanin 7
191	MOAP1	NM_022151	modulator of apoptosis 1
192	FRMD4B	NM_015123	FERM domain containing 4B
193	LOC100128288	NR_024447	uncharacterized LOC100128288
194	SOX18	NM_018419	SRY-box 18
