# Supplementary material for: Analysis of Under-Diagnosed Malignancy during Fine Needle Aspiration Cytology of Lymphadenopathies
Source: Int J Mol Sci. 2023 Aug 3;24(15):12394. doi: 10.3390/ijms241512394 (PMC10418811; doi:10.3390/ijms241512394)
Supplement: Supplementary file 1 [file ijms-24-12394-s001.zip › Table S3. 65 candidate genes used in cBioPortal.pdf]

Table S3. 65 candidate genes used in cBioPortal.

	Genes	Identifier	Description
1	FOS	NM_005252	Fos proto-oncogene, AP-1 trancription factor subunit
2	PMPCB	NM_004279	peptidase, mitochondrial processing beta subunit
3	GHITM	NM_014394	growth hormone inducible transmembrane protein
4	HDAC10	NM_032019	histone deacetylase 10
5	RASA4B	NM_001277335	RAS p21 protein activator 4B
6	CAMLG	NM_001745	calcium modulating ligand
7	AP3S1	NM_001284	adaptor related protein complex 3 sigma 1 subunit
8	PPIP5K1	NM_014659	diphosphoinositol pentakisphosphate kinase 1
9	C2orf69	NM_153689	chromosome 2 open reading frame 69
10	ENDOG	NM_004435	endonuclease G
11	PRKAR2B	NM_002736	protein kinase cAMP-dependent type II regulatory subunit beta
12	TRIP10	NR_110231	thyroid hormone receptor interactor 10
13	TTC4	NM_004623	tetratricopeptide repeat domain 4
14	FPR1	NM_001193306	formyl peptide receptor 1
15	N6AMT2	NM_174928	EEF1AKMT1, EEF1A Lysine Methyltransferase 1
16	TMEM167A	NM_174909	transmembrane protein 167A
17	RAB28	NM_001159601	RAB28, member RAS oncogene family
18	FAM189B	NM_198264	family with sequence similarity 189 member B
19	CD163	NM_203416	CD163 molecule
20	TICAM1	NM_182919	toll like receptor adaptor molecule 1
21	G0S2	NM_015714	G0 /G1 switch 2
22	NCAPD3	NM_015261	non-SMC condensin II complex subunit D3
23	LINC01138	NR_104014	long intergenic non-protein coding RNA 1138
24	HMMR	NM_001142556	hyaluronan mediated motility receptor
25	LINC00623	NR_024511	long intergenic non-protein coding RNA 623
26	PPP6R2	NM_001242900	protein phosphatase 6 regulatory subunit 2
27	C9orf114	NM_016390	SPOUT1, chromosome 9 open reading frame 114
28	MRPS10	NM_018141	mitochondrial ribosomal protein S10
29	LRRK1	NM_024652	leucine-rich repeat kinase 1
30	FABP4	NM_001442	fatty acid binding protein 4
31	ATP5L2	NM_001165877	ATP5MGL, ATP synthase, H+ transporting, mitochondrial Fo complex subunit G2

32	DNASE1	NM_005223	deoxyribonuclease I
33	HSF1	NM_005526	heat shock transcription factor 1
34	PSMB4	NM_002796	proteasome subunit beta 4
35	SGK1	NM_001291995	serum /glucocorticoid regulated kinase 1
36	LINC00869	NR_111953	long intergenic non-protein coding RNA 869
37	MCOLN2	NM_153259	mucolipin 2
38	TRA2B	NM_004593	transformer 2 beta homolog (Drosophila)
39	FILIP1L	NM_001282794	filamin A interacting protein 1-like
40	RBM41	NM_001171080	RNA binding motif protein 41
41	BRPF1	NM_004634	bromodomain and PHD finger containing 1
42	TSEN34	NM_001282333	tRNA splicing endonuclease subunit 34
43	MEF2B	NM_001145785	myocyte enhancer factor 2B
44	TOR1A	NM_000113	torsin family 1 member A
45	PPP2R4	NM_021131	PTPA, protein phosphatase 2A regulatory subunit 4
46	ELOVL5	NM_021814	ELOVL fatty acid elongase 5
47	MEF2BNB-MEF2B	NR_027307	BORCS8-MEF2B, transcript variant 2, non-coding RNA
48	TBC1D22A	NM_001284303	TBC1 domain family member 22A
49	TMEM64	NM_001008495	transmembrane protein 64
50	LSM1	NR_045492	LSM1 homolog, mRNA degradation associated
51	BANF1	NM_001143985	barrier to autointegration factor 1
52	MYB	NM_005375	MYB proto-oncogene, transcription factor
53	IVNS1ABP	NM_006469	influenza virus NS1A binding protein
54	NDUFA6	NM_002490	NADH:ubiquinone oxidoreductase subunit A6
55	MRPS6	NM_032476	mitochondrial ribosomal protein S6
56	BAG1	NM_001172415	BCL2 associated athanogene 1
57	SMC1A	NM_001281463	structural maintenance of chromosomes 1A
58	RFX7	NM_022841	regulatory factor X7
59	CLHC1	NM_152385	clathrin heavy chain linker domain containing 1
60	DAZAP1	NM_170711	DAZ associated protein 1
61	PPP1R12C	NM_017607	protein phosphatase 1 regulatory subunit 12C
62	EIF4G1	NM_198241	eukaryotic translation initiation factor 4 gamma 1
63	COX7A2L	NM_004718	cytochrome c oxidase subunit 7A2 like
64	MRPL21	NM_181514	mitochondrial ribosomal protein L21

65	PHOX2A	NM_005169	paired like homeobox 2a
