# Supplementary material for: Analysis of Under-Diagnosed Malignancy during Fine Needle Aspiration Cytology of Lymphadenopathies
Source: Int J Mol Sci. 2023 Aug 3;24(15):12394. doi: 10.3390/ijms241512394 (PMC10418811; doi:10.3390/ijms241512394)
Supplement: Supplementary file 1 [file ijms-24-12394-s001.zip › Table S4. Details of primers used for qRT-PCR analysis.pdf]

Table S4. Details of primers used for qRT-PCR analysis.

Gene name		Primer Sequences	Accession No.	Product size (bp)
ENDOG	(F)	CACCTCAACCAGAATGCCTGGA	NM_004435.2	129
	(R)	GTAGGATTTCCCATCAGCCTCTG		
FOS	(F)	TACTACCACTCACCCGCAGA	NM_005252	474
	(R)	CAGGTTGGCAATCTCGGTCT		
HDAC10	(F)	TGTGTGTTCAACAACGTGGC	NM_032019	367
	(R)	CTGAGTCAAATCCTGCCGAGA		
HMMR	(F)	GGCTGGGAAAAATGCAGAGGATG	NM_001142556.2	110
	(R)	CCTTTAGTGCTGACTTGGTCTGC		
PMPCB	(F)	AGTGGACTCAGAGTAGCTTCGG	NM_004279.3	121
	(R)	GCTCCAGAAAGTGTGCTGTTCC		
PRKAR2B	(F)	AACCGATTCACAAGGCGTGCCT	NM_002736.3	144
	(R)	CAGCAGGATGTCTTTGCAAGCC		
GAPDH	(F)	AAGGACTCATGACCACAGTC	NM_001289745.2	160
	(R)	TTCAGCTCAGGGATGACCTT		
